# Editorial Comment: Radical prostatectomy without prior biopsy following multiparametric magnetic resonance imaging and prostate-specific membrane antigen positron emission tomography

**DOI:** 10.1590/S1677-5538.IBJU.2023.01.06

**Published:** 2023-01-26

**Authors:** Saulo Borborema Teles, Rafael Sanchez-Salas, Arie Carneiro

**Affiliations:** 1 Hospital Israelita Albert Einstein Departamento de Urologia São Paulo SP Brasil Departamento de Urologia, Hospital Israelita Albert Einstein, São Paulo, SP, Brasil; 2 McGill University Division of Urology Department of Surgery Montreal Quebec Canada Department of Surgery, Division of Urology, McGill University, Montreal, Quebec, Canada

Valentin H. Meissner^1^, Isabel Rauscher^2^, Kristina Schwamborn^3^, Jan Neumann^4^, Gregor Miller^5^, Wolfgang Weber^2^, et al.

**Eur Urol. 2022 Aug;82(2):156-160.**

**DOI: 10.1016/j.eururo.2021.11.019 | ACCESS: 34887117**

## COMMENT

Recently published in European Urology, Meissner et al (1) presented a series of 25 patients undergoing radical prostatectomy without performing a prostate biopsy, basing the surgical approach on the combination of multiparametric prostate resonance imaging (mpMRI) and prostate-specific membrane antigen positron emission tomography (PET-PSMA). Using the criterion of PIRADS≥4 and PET-Score≥4, 25 patients presented 100% of ISUP≥2. Despite the result found, we must be aware of the various weaknesses of the study and the potential repercussions that may be erroneously considered.

The aforementioned study is a retrospective analysis, with a small number of patients, and some ethical issues to consider. The approach certainly shows the potential of imaging risk stratification but simultaneously falls into the controversial field of cancer treatment without pathology support and patient exposure to the morbidity of radical prostatectomy. Although it was described that the patient was extensively instructed on the need for a prostate biopsy to better understand the disease and, then, to define the available therapeutic options (active surveillance, focal therapy, surgery, or radiotherapy), the surgical procedure was performed without the biopsy. Our biggest concern is that some urologists, considering these data, may indicate surgery without biopsy in clinical practice without a research protocol and an approved informed consent.

In those patients in which radiotherapy was chosen, it was strictly necessary to perform a prostate biopsy to define the applied dose and evaluate the association with hormonal therapy. However, the same need also applies to the surgical approach, as it is paramount to define whether or not to perform extended lymphadenectomy, a procedure with considerable morbidity, and the criterion used for its performance is not described in the study.

To decide whether or not to perform lymphadenectomy, we still use clinic-pathological nomograms associated with MRI to help us in the decision. There is a tendency to perform less lymphadenectomy because its oncological role has been questioned (2, 3), but we still do not have data that support that PET-PSMA can replace these nomograms. In this way, patients may be undertreated by not performing lymphadenectomy when guided only by imaging exams.

In the supplementary material presented by the article, the author described that the sensitivity and positive predictive value (PPV) of mpMRI are 37% and 81%, respectively, and PET-PSMA has a sensitivity of 38% and a PPV of 81%. Using the combination of images, the sensitivity increased to 41% and PPV reached 80%. The author also described that sensitivity and PPV are slightly reduced in the lesion-based analysis using the criteria of PIRADS≥4 and PET-Score≥4. These numbers demonstrate the possibility of failure, both in the detection of the disease, with the loss of a patient with clinically significant prostate cancer (csPCa) and the possibility of failure in the diagnosis, with a chance of finding a lesion that would not require treatment or be a candidate for a less morbid approach, leading to over-treatment.

A recent meta-analysis published by Satapathy (4) shows that PET-PSMA sensitivity, specificity, positive likelihood ratio, and negative likelihood ratio for detection of csPCa were 0.99 (95% CI, 0.88– 1.00), 0.49 (95% CI, 0.36–0.62), 1.9 (95% CI, 1.5–2.5), and 0.02 (95% CI, 0.00–0.28), respectively. Considering a subgroup of initial detection of csPCa (ISUP>2), the study presents 1 case of a false negative in a population of 25 patients and the false positive rate varies between 4 and 50% in the included studies.

According to Emmet et al (5), the combination of MRI+PSMA showed that of 129 patients with a non-clinically significant tumor, the combination of imaging exams demonstrated a positive lesion in 78 patients (60%). The sensitivity of the combination of tests was 97%, specificity 40%, PPV 67%, and negative predictive value (NPV) 91%. The false negative rate was high, 17% on MRI and 10% on PSMA. Eiber et al (6) showed that the accuracy of the combination of MRI+PET-PSMA was 88%, sensitivity 76%, and specificity 97%. Thus, it is a consensus in the current literature that the MRI+PET-PSMA combination still does not have sufficient strength to determine csPC lesions.

Scheltema et al (7) performed a study using the mpMRI PIRADS 4–5 and PET-PSMA combination in 56 patients, the sensitivity, specificity, NPV, and PPV were 92%, 90%, 96%, and 81%, respectively. These percentages are based on patients already diagnosed with intermediate and high-risk prostate cancer. An approach with a combination of these imaging tests in patients without the diagnosis would most likely have lower rates.

The authors report that 100% of the patients had a csPCa, however, as it is a retrospective study with a low number of patients, it is difficult to understand important flaws in the selection criteria of the included patients. It would only be possible to draw some conclusions by studying the entire sample of the service with the same profile as the patients studied in this paper.

There are a few urological tumors treated without previous biopsy. Among them, we can mention adrenal, kidney, and testicular neoplasm. In these cases, image analysis has high accuracy and a low false positive rate for tumor detection. The delay in diagnosis can negatively impact patient survival and the result would not change the technical approach. In addition, performing the biopsy may alter the tumor staging or there is a risk of dissemination through the biopsy puncture site. All these criteria do not apply to prostate cancer and surgery may exposes patients to a negative impact on quality of life.

The prostate biopsy provides more information about the aggressiveness of the tumor, making possible a more conservative approach, whether by active surveillance or more recently partial gland ablation with well-known side effects such as sepsis (<1.5%), urinary retention (<2%), or hematuria requiring catheterization (<1%). Currently, lower complication rates are seen with the transperineal approach (8).

Currently, the challenge is to understand tumor biology, using analyzes such as Decipher, Oncotype, and other tests, to understand which patients, have cancer-specific survival benefits and may have an advantage from local treatment and to give a personalized approach. The proposal of aggressive local treatment of prostate cancer is opposed to the current search for lower morbidity treatment options.

We have been rapidly evolving in the diagnostic methods deployed in the prostate cancer pathway. It is our humble belief that imaging is not yet accurate enough to indicate radical treatment based on the combination of mpMRI+PET-PSMA information.

We commend Meissner et al (1) for their broad vision and motivation to explore novel approaches, but in this scenario, we would first need a study with a rigid methodology comparing radiological findings with both biopsy and final pathology. This could indeed create a more reliable clinical path to eventually offer radical prostatectomy without biopsy.
